# Spanish is not just one: A dataset of Spanish dialect recognition for LLMs

**DOI:** 10.1016/j.dib.2025.112088

**Published:** 2025-09-18

**Authors:** Gonzalo Martínez, Marina Mayor-Rocher, Cris Pozo Huertas, Nina Melero, María Grandury, Pedro Reviriego

**Affiliations:** a Information Processing and Telecommunications Center (IPTC), Universidad Politécnica de Madrid, Avda. Complutense 30, 28040 Madrid, Spain; b Facultad de Filosofía y Letras, Universidad Autónoma de Madrid, c/ Francisco Tomás y Valiente 1, 28049 Madrid, Spain; c New York University, Madrid Campus, C. del Barquillo, 13, 28004 Madrid, Spain; d SomosNLP, Spain

**Keywords:** Natural language processing, Large language models, Evaluation, Machine learning, Spanish dialects, Linguistics, AI, Language variation

## Abstract

This paper presents a dataset designed to assess the capability of Large Language Models (LLMs) in handling different Spanish dialects. While multilingualism is widely recognized as a crucial aspect of NLP, dialectal evaluation remains largely unexplored. Spanish, spoken by over 600 million people, exhibits significant lexical, morphological, and syntactic variation across regions. Recognizing these linguistic and cultural differences is essential for preserving smaller dialects, preventing their marginalization, and ensuring that Spanish is not reduced to a monolithic language. To address this gap, we introduce a dataset specifically designed to analyze whether LLMs can accurately identify different Spanish varieties while also measuring their potential preference for specific dialects. The dataset consists of 30 carefully crafted multiple-choice questions, requiring models to select the most appropriate option from different regional variations. Each question has been meticulously developed and reviewed by linguistic experts, undergoing multiple refinement cycles to ensure linguistic accuracy and effectiveness in detecting dialectal biases. This dataset represents an important step toward developing more inclusive and fair evaluation frameworks for Spanish Natural Language Processing (NLP). By identifying potential biases in LLMs and analyzing their ability to adapt to regional linguistic variations, this work contributes to the broader goal of equitable language representation in AI-driven text generation and comprehension tasks.

Specifications Table

**Table:** 

Subject	Computer Science
Specific subject area	Artificial Intelligence
Type of data	Table in a csv or xlsx file
Data collection	The questions were written by a linguistic expert to reflect morphosyntactic and lexical variations across Spanish-speaking regions. A revision process was implemented to ensure the quality and clarity of the questions. Two additional linguistic experts examined each question for linguistic accuracy and cultural appropriateness. Revisions were made collaboratively with the original creator to refine the questions while ensuring accessibility and unambiguous interpretation. Finally, the questions were tested on various LLMs to verify that they understood the tasks and could highlight potential dialectal biases effectively.
Data source location	The data was collected in Madrid, Spain.
Data accessibility	Repository name: It's the same but not the same: Do LLMs distinguish Spanish varieties? Data identification number: DOI: 10.5281/zenodo.15101403 Direct URL to data: https://zenodo.org/records/15101403
Related research article	It's the same but not the same: Do LLMs distinguish Spanish varieties? https://arxiv.org/abs/2504.20049 Accepted for publication in the journal “*Procesamiento del Lenguaje Natural*”

## 1 Value of the Data

• The dataset is specifically designed to evaluate the knowledge of Spanish dialects that LLMs have.
• It has already been used to analyse LLM knowledge of linguistic variations and performance across different Spanish dialects in our work previous work [1].
• The questions are tailored to reflect regional differences in vocabulary, grammar, and usage.
• The dataset has been created and reviewed by three experts in linguistics and Spanish language variation.
• The dataset can also be used for human evaluation, both of native speakers to detect their dialect or of Spanish students to make them aware of the different varieties of Spanish.
• The dataset can also be used in sociolinguistic research to study the use of dialects depending on the social class or education level.

## 2 Background

Spanish is the world’s second-most spoken native language, yet English has historically led Artificial Intelligence (AI) language processing. Large Language Models (LLMs) exhibit remarkable capabilities [2,3], however evaluating their Spanish performance poses unique challenges. The Massive Multitask Language Understanding (MMLU) benchmark, for example, features questions derived from English-oriented exams like the GRE (Graduate Record Examination) and US Medical Licensing tests [4,5]. Although these tests gauge broad knowledge, they do not account for the diverse dialects and linguistic norms intrinsic to Spanish.

Spanish encompasses distinct variants, each with specialized vocabularies, grammar, and cultural references; the traditional linguistic macroareas define the following varieties: Andean (Bolivia, Ecuador and Peru), Antillean (Cuba, Dominican Republic and Puerto Rico), Chilean, Continental Caribbean (Colombia and Venezuela), Mexican and Central American (Costa Rica, El Salvador, Guatemala, Nicaragua, Panama and Mexico), European Peninsular (Spain), and Rioplatense (Argentina, Paraguay and Uruguay). Directly translating English tests into “generic” Spanish can overlook such nuances, leading to biased or incomplete evaluations. Observations suggest that some advanced language models are able to discern some dialectal subtleties, while many others conflate regional differences. Hence, there is a pressing need for Spanish-specific benchmarks that capture the full range of Spanish usage, ensuring fair and accurate assessment of LLMs across all Spanish-speaking regions.

## 3 Data Description

The data is a set of 30 multiple-choice questions whose main objective is to assess the ability of different language models (LLMs) to use different varieties of Spanish. The number of questions was set by making a compromise between the effort needed to elaborate and run the dataset and the goal of providing a comprehensive evaluation. The questions cover aspects such as order of syntactic constituents in a sentence, lexical variation, and the use of different verb forms. Table 1, Table 2, Table 3

**Table 1:** Example of a question.

¿Cuál suena más natural? / Which one sounds more natural?	
A. «Llegas tarde, vístete y corre».	(Additional note: Peninsular, Chilean Spanish, among others.)
B. «Llegas tarde, vístete y córrele».	(Additional note: Mexican Spanish.)

**Table 2:** Example of a question.

¿Qué verbo usas para describir la acción de ponerse de pie? / What verb do you use to describe the action of standing up?	
A. levantarse	(Additional note: Peninsular Spanish.)
B. pararse	(Additional note: Antillean, Mexican Spanish, among others.)

**Table 3:** Example instructions.

«Eres de Centroamérica, nacido en México, Guatemala, Costa Rica, Honduras, Nicaragua, Panamá o El Salvador. Responde a la siguiente pregunta con la opción que te resulte más natural. Ajústate solo a las opciones dadas.»/ «You are from Central America, born in Mexico, Guatemala, Costa Rica, Honduras, Nicaragua, Panama, or El Salvador. Answer the following question with the option that feels most natural to you. Stick only to the options provided.»

As an example, the following question, which looks at the addition of the exhortative enclitic pronoun “le” to the verb “corre”, is shown:

Similarly, questions are posed that require deciding between “levantarse” or “pararse” to describe the action of standing up, depending on the variety of Spanish with which the model identifies:

These questions have been designed by a team of expert linguists and are intended to assess the ability of language models to distinguish and reproduce the morphosyntactic and lexical peculiarities of seven varieties of Spanish: Andean, Antillean, Chilean, Continental Caribbean, Mexican and Central American, European Peninsular, and Rioplatense.

This test can be applied to assess which Spanish dialect is used by default by a given LLM. In this case, the questions are asked directly without assigning any role to the LLM. To evaluate the knowledge of a specific dialect, a prompt that instructs the model to behave like a native speaker of a specific region is used. For example:

The format of the elements of the dataset is summarized in Table 4. The fields include the type of question, the instructions on the role of the LLM as a native speaker of one of the Spanish varieties and the question itself. The questions are multiple choice and designed to support a variable number of possible and correct options. This is achieved by having a list of possible options, then the options and finally the list of correct options.

**Table 4:** Fields of the dataset elements.

Field	Description and values
**Question type**	Type of the question: - Lexical Variation (0) - Morphosyntactic (1)
**Instructions**	Instructions on the role of the LLM to answer as a native speaker of the: - Andean (Bolivia, Ecuador and Peru) - Antillean (Cuba, Dominican Republic and Puerto Rico) - Chilean (Chile) - Continental Caribbean (Colombia and Venezuela) - Mexican and Central American (Costa Rica, El Salvador, Guatemala, Nicaragua, Panama and Mexico) - European Peninsular (Spain) - Rioplatense (Argentina, Paraguay and Uruguay) Spanish varieties.
**Question**	Text with the multiple-choice question.
**Possible options**	List of the potential options as a list with the format: A,B,C,D,… This is intended to support different number of options for each question. In the datasets some questions have only two options (A,B) while others have up to eight (A,B,C,D,E,F,G,H).
**Options**	The text for each of the possible options.
**Correct options**	Subset of the possible options that are correct.

The dataset has been designed such that the questions and possible options are the same for all Spanish varieties. The variety only determines the instructions given to the LLM in terms of acting as a native speaker of that variety and the list of correct options. This means that all varieties are evaluated on the same questions which makes the results more easily comparable and also enables the testing of the default variety used by an LLM by just removing the instructions not assigning it a role for any variety.

## 4 Experimental Design, Materials and Methods

The objective was to create a publicly available dataset of multiple-choice questions designed to assess the biases of Large Language Models (LLMs) toward different varieties of Spanish. The methodology used to create this dataset is the following:

• **Question Development:** A linguistic expert initially created a set of multiple-choice questions targeting different Spanish dialects. These questions focus on elements like order of syntactic constituents in a sentence, word choices, and verb usage that highlight morphosyntactic and lexical variations across Spanish-speaking regions. Their overall purpose is to reveal any potential bias in how LLMs handle diverse dialectal features.
• **Peer Review**: Two additional linguistic experts examined each question to ensure clarity, cultural appropriateness, and linguistic accuracy. Any revisions were agreed upon through collaboration with the original question creator, refining each item to effectively highlight dialectal biases while remaining accessible and unambiguous for respondents.
• **Refinement:** The final set of questions was then tested on various LLMs to ensure they were capable of understand the tasks of the different questions.

## Limitations

The dataset represents a new step in dialect evaluation and may not be optimal for assessing artificial intelligence models. On the other hand, the dataset is composed exclusively of questions about variation in morphology, syntax, and lexicon, excluding other types of variation such as pragmatics or discourse-level features like reading comprehension. Finally, the number of questions is limited to 30 (20 that target grammar, 10 that target vocabulary)*.*

## Ethics Statement

The data set does not involve human subjects, animal experiments, or any data collected from social media platforms.

## CRediT Author Statement

**Gonzalo Martínez:** conceptualisation and writing. **Marina Mayor-Rocher:** conceptualisation, data creation and curation, methodology, writing and review. **Cris Pozo Huertas:** conceptualisation, data creation and curation, methodology, writing and review. **Nina Melero:** conceptualisation, data creation and curation, methodology, writing and review. **María Grandury:** methodology, writing and conceptualisation. **Pedro Reviriego:** conceptualisation, methodology, writing, project coordination.

## Data Availability

ZenodoIt's the same but not the same: Do LLMs distinguish Spanish varieties? (Original data)
